# Functional Heterogeneity of Mouse and Human Brain OPCs: Relevance for Preclinical Studies in Multiple Sclerosis

**DOI:** 10.3390/jcm9061681

**Published:** 2020-06-02

**Authors:** Ana Bribián, Eva M. Medina-Rodríguez, Fernando Josa-Prado, Isabel García-Álvarez, Isabel Machín-Díaz, Pedro F. Esteban, Verónica Murcia-Belmonte, Lorena Vega-Zelaya, Jesús Pastor, Leoncio Garrido, Fernando de Castro

**Affiliations:** 1 Instituto Cajal-CSIC, CSIC, Avda. Dr. Arce 37, 28002 Madrid, Spain; anabribian@gmail.com (A.B.); fjosa@cajal.csic.com (F.J.-P.); 2 Grupo de Neurobiología del Desarrollo-GNDe, Hospital Nacional de Parapléjicos, Finca “La Peraleda” s/n, 45071 Toledo, Spain; emm220@med.miami.edu (E.M.M.-R.); imachin@sescam.jccm.es (I.M.-D.); pfesteban@sescam.jccm.es (P.F.E.); vmurcia@umh.es (V.M.-B.); 3 Facultad de Ciencias Experimentales, Universidad Francisco de Vitoria, Ctra. Pozuelo-Majadahonda Km 1800, Pozuelo de Alarcón, 28223 Madrid, Spain; isabel.alvarez@ufv.es; 4 Instituto de Neurociencias, Universidad Miguel Hernández-CSIC, Campus San Juan de Alicante, 03550 Alicante, Spain; 5 Neurofisiología Clínica; Instituto de Investigación Sanitaria, Hospital Universitario de la Princesa, 28006 Madrid, Spain; lorenacarolina.vega@salud.madrid.org (L.V.-Z.); jesus.pastor@salud.madrid.org (J.P.); 6 Instituto de Ciencia y Tecnología de Polímeros (ICTP-CSIC), CSIC, Juan de la Cierva 3, 28006 Madrid, Spain; lgarrido@cetef.csic.es

**Keywords:** oligodendrocyte, myelin, multiple sclerosis, human, remyelination, leukodystrophies, demyelination

## Abstract

Besides giving rise to oligodendrocytes (the only myelin-forming cell in the Central Nervous System (CNS) in physiological conditions), Oligodendrocyte Precursor Cells (OPCs) are responsible for spontaneous remyelination after a demyelinating lesion. They are present along the mouse and human CNS, both during development and in adulthood, yet how OPC physiological behavior is modified throughout life is not fully understood. The activity of adult human OPCs is still particularly unexplored. Significantly, most of the molecules involved in OPC-mediated remyelination are also involved in their development, a phenomenon that may be clinically relevant. In the present article, we have compared the intrinsic properties of OPCs isolated from the cerebral cortex of neonatal, postnatal and adult mice, as well as those recovered from neurosurgical adult human cerebral cortex tissue. By analyzing intact OPCs for the first time with 1H High Resolution Magic Angle Spinning Nuclear Magnetic Resonance (1H HR-MAS NMR) spectroscopy, we show that these cells behave distinctly and that they have different metabolic patterns in function for their stage of maturity. Moreover, their response to Fibroblast Growth Gactor-2 (FGF-2) and anosmin-1 (two molecules that have known effects on OPC biology during development and that are overexpressed in individuals with Multiple Sclerosis (MS)) differs in relation to their developmental stage and in the function of the species. Our data reveal that the behavior of adult human and mouse OPCs differs in a very dynamic way that should be very relevant when testing drugs and for the proper design of effective pharmacological and/or cell therapies for MS.

## 1. Introduction

Oligodendrocyte Precursor Cells (OPCs) are the sole source of oligodendrocytes, the myelin-forming cells in the Central Nervous System (CNS), and they are widely distributed throughout both gray and white matter [1,2,3,4,5]. The vast majority of mature oligodendrocytes are born during postnatal life but in adults, oligodendrocytes can also be generated from OPCs that continue to divide and generate new-myelinating cells during adulthood [5,6,7,8,9,10,11,12]. In the adult brain, OPCs constitute 2–3% of cells in gray matter and 5–8% in white matter [1]. These cells are maintained as a resident population by self-renewal, representing the main proliferative cell type outside the neurogenic regions of the CNS [1,7,12,13,14]. However, the adult OPC population is not homogeneous as OPCs cycle less intensely with age, their proliferation rate is higher in white matter than in the gray matter, and their differentiation rate declines progressively in postnatal and adult life [1,5,8,9,10,11,15,16]. Although the functions of adult-born oligodendrocytes remain largely unclear, they apparently maintain physiological oligodendroglial homeostasis and under pathological circumstances, they mediate long-term repair in white matter after injury and disease, even in MS where they can partially restore the lost myelin sheaths [12,17,18,19]. In demyelinating diseases, there is an up-regulation of different molecules in the microenvironment in and around the lesions, including several factors that are important for developmental oligodendrogliogenesis, such as secreted semaphorins, growth factors, mitogens, and adhesion molecules [20,21,22,23,24,25,26].

In the present study, we focused on cerebral cortex OPCs to avoid any variability introduced by anatomical origin or location. We examined the degree of intrinsic heterogeneity (both heterochronic and interspecies) of these cortical OPCs and we described the dynamic changes in their intrinsic properties when assessed by Nuclear Magnetic resonance (NMR) (i.e, metabolic patterns), and how these changes affect their response to two molecules known to influence OPC biology during development and remyelination: Fibroblast growth Factor-2 (FGF-2) and anosmin-1 [12,21,27,28,29,30,31,32,33,34,35,36]. We systematically studied mouse cortical OPCs at different ages and OPC isolated from the human adult cerebral cortex obtained from neurosurgery biopsies (resection margins of tumors and/or epilepsy surgery [37]). In terms of the latter, the scarcity of articles dealing with human OPCs is especially remarkable [23,37,38,39,40,41].

The data we obtained highlight the intrinsic physiological differences between murine OPCs isolated at different ages. Indeed, while OPC proliferation and migration were enhanced similarly by FGF2, this factor produced different effects on OPC differentiation. By contrast, anosmin-1 elicited diverse effects on differentiating, and especially on migrating, OPCs, yet not on the proliferation of these cells. While FGF2 was a chemoattractant for human and murine OPCs, anosmin-1 only attracted OPCs isolated from tumor samples and repelled those from non-tumoral samples. Neither FGF2 nor anosmin-1 affected human adult OPC differentiation. These observations confirm the chronological and inter-species heterogeneity of OPCs, adversity that will probably be relevant when designing effective pharmacological and/or cell therapies to repair myelin loss in MS and other demyelinating diseases.

## 2. Materials and Methods

### 2.1. Animals

CD-1 mice were obtained from (Charles River Laboratories, Inc., Wilmington, MA) and maintained in the animal facilities of the Hospital Nacional de Parapléjicos (Toledo, Spain). OPCs were obtained from differently aged animals: embryonic (E16); postnatal (P0, P7, P15); and adult (P60). All experimental procedures were carried out in accordance with Spanish (RD223/88) and European (2010/63/EU) guidelines, and were approved by the Animal Review Board at the Hospital Nacional de Parapléjicos (SAPA001). 

### 2.2. Human Biopsies

Biopsies of human tumor (resection of the safety margins but not the tumor itself) and non-tumor tissue (epilepsy surgery) were obtained from the Neurosurgery Service at the Hospital La Princesa (Madrid, Spain). All human samples were obtained from the adult brain cortex and the biopsies were predominantly from the temporal lobe (Table 1). They were transported at 4 °C as rapidly as possible in AQIX^®^ RS-I (AQIX Ltd., Imperial College BioIncubator) to reduce cell damage. All subjects gave their informed consent for inclusion before they participated in the study. The study was conducted in accordance with the guidelines, and the protocol was approved by the Research Ethics Committee of the Hospital Nacional de Parapléjicos de Toledo (87/2012) and the Instituto Cajal–CSIC (440/2016 and 2016/049/CEI3/20160411). 

### 2.3. HOG Cells

An immortalized Human OligodendroGlioma cell line (HOG), was grown at 37 °C in an atmosphere of 5% CO_2_, and in Dulbecco’s Modified Eagle’s Medium (DMEM, Gibco|Thermo Fisher Scientific, Waltham, MA, USA) supplemented with 10% Fetal Bovine Serum (FBS, Gibco|Thermo Fisher Scientific, Waltham, MA, USA), 100 U/mL penicillin and 100 µg/mL streptomycin (Sigma-Aldrich, San Luis, AZ, USA). The HOG cell line, established from a surgically removed human oligodendroglioma was kindly provided by Dr. A. T. Campagnoni (University of California, UCLA, Los Angeles, CA, USA) via Dr. J.A. López-Guerrero (Universidad Autónoma de Madrid, Madrid, Spain).

### 2.4. Oligodendrocyte Culture

OPCs were isolated as described previously [19,34,38]. Briefly, the cerebral cortex of mice aged E16, P0, P7, P15, and P60 was dissected out and the meninges were carefully removed. The cortical tissue was mechanically disaggregated and it was then enzymatically dissociated in a papain solution, filtered using a 100 µm nylon mesh strainer (BD Biosciences, Franklin Lakes, NJ, USA), and seeded in polyornithine-treated flasks in DMEM containing 10% FBS (BioWhittaker | Lonza, Basiela, Switzerland) and an antibiotic anti-mycotic solution (100 U/mL penicillin/0.1 mg/mL streptomycin and 0.25 µg/mL Amphotericin B; Sigma-Aldrich, San Luis, AZ, USA). The cultures were maintained at 37 °C and in 5% CO_2_, and the medium was changed every 4 days, adding 10 ng/mL of human PDGF-AA (Millipore, Burlington, NJ, USA) to the cultures from adult mice. When the cultures reached confluence, they were shaken overnight at 250 rpm to detach the oligodendrocyte progenitors growing on top of the confluent astrocyte monolayer. The medium was then filtered through a 40 μm nylon mesh strainer (BD Biosciences, Franklin Lakes, NJ, USA) and centrifuged at 900 rpm. The recovered cells were seeded twice (45 min each) in bacterial grade Petri dishes (Sterilin Containers|Thermo Fisher Scientific, Waltham, MA, USA) to remove the adherent microglia and after another round of centrifugation, the resulting pellet was resuspended and the OPCs were counted and seeded.

For cells obtained from human biopsies, the same protocol was used with certain modifications as published previously [37]. This protocol allowed us to obtain sufficient human OPCs to perform the necessary assays. Briefly, rather than 75 cm^2^ flasks, 25 cm^2^ flasks were used with a final volume of 5 mL rather than 10 mL OPC medium per flask. In addition, the medium was supplemented with 10 ng/mL of human PDGF-AA (Millipore, Burlington, NJ, USA), as in the cultures obtained from adult mice. Cultures reached confluence after 30–40 days and the flasks were shaken at 230 rpm instead of 250 rpm.

### 2.5. Preparation of Extracts for Anosmin-1

Proteins from the extracellular matrix (ECM) of CHO cells (CT) and CHO cells stably transfected with an expression plasmid carrying a C-terminal hemaglutinin-tagged (HA) version of the human KAL1 cDNA (A1 [42]) were extracted as described previously [32]. Briefly, the cells were washed once with calcium/magnesium-free Hank’s Balanced Salt Solution (Gibco|Thermo Fisher Scientific, Waltham, MA, USA) and they were then incubated at 4 °C for 30 min in a gently rocking 10 cm diameter culture dish in 1 mL of 20 mM phosphate buffer (PB, pH 7.4), containing 350 mM NaCl and complete EDTA free protease inhibitor (Roche, Basiela, Switzerland). The ECM proteins released into the buffer were concentrated ten-fold with Amicon Ultra-4 Ultracel-30k (Millipore Corporation, Burlington, NJ, USA) and used in the chemotaxis experiments (ECM extracts). The presence of anosmin-1 was confirmed in Western blots probed with a peroxidase-conjugated rat monoclonal antibody against HA (High Affinity 3F10; Roche, Basiela, Switzerland). Equal amounts of protein from untransfected and transfected cells were used in all the in vitro experiments described below.

### 2.6. Chemotaxis

To test chemoattraction, cells (murine and human OPCs—isolated from non-tumoral (haOPCs) and tumoral (malOPCs) samples—and HOG cells) were seeded in the upper chamber of a chemotaxis chamber (40,000 cells/transwell), and FGF2 (20 ng/mL; R&D Systems, Minneapolis, MN, USA) or 0.1 mg/mL of CT and A1 cell extracts (see below) were added to the lower chamber in Bottenstein-Sato (BS) medium, as reported previously [21,29,34,43]. After 20 h at 37 °C, the cells were fixed in 4% paraformaldehyde (PFA) for 10 min at room temperature and the OPCs were stained with antibodies against Ganglioside GT3 (A2B5, 1:20; Developmental Studies Hybridoma Bank, IA, US) and the Olig-2 oligodendroglial transcription factor (1:200, Millipore, Burlington, NJ, USA), and HOG cells were stained with Hoechst. After immunostaining, the filters were examined using an In Cell Analyzer 1000 (GE-HealthCare, Chicago, IL, USA) and 15–20 microphotographs from each membrane were taken randomly. The In Cell Analyzer 1000 Work station software was used (GE-HealthCare, Chicago, IL, USA) to quantify the number of transmigrated OPCs per field, expressing the data as the percentage of migrating OPCs relative to the controls ± s.e.m. (Considered as 100% [34]).

### 2.7. Time-Lapse Imaging

For a detailed study of OPC migration, time-lapse video-microscopy (Leica, DMI 6000B) was performed to observe the behavior of the cortical OPCs. Purified OPCs were placed on poly-l-lysine and laminin coated coverslips (10,000 cells/coverslip; Sigma-Aldrich, San Luis, AZ, USA) and FGF2 (20 ng/mL; R&D Systems, Minneapolis, MN, USA) or CT CHO or A1-expressing CHO cell extracts (0.1 mg/mL, see above) were added in BS medium. Images were taken every 20 min for 18 h and the speed of cell motility was assessed with the manual tracking plugin of the ImageJ software. The values represent the ratio of the number of stops (times distance measured was equal to 0) over the number of cells.

### 2.8. Differentiation Assay

Purified OPCs were placed on coverslips coated with poly-l-lysine and laminin (20,000 cells/coverslip), and FGF2 (20 ng/mL; R&D Systems, Minneapolis, MN, USA) or total protein from CT CHO cells or A1-expressing CHO cells (0.1 mg/mL, see above) were added in differentiation medium (BME:F12 media (Gibco | Thermo Fisher Scientific, Waltham, MA USA) [1:1] supplemented with 100 μg/mL transferrin, 20 μg/mL putrescine, 12.8 ng/mL progesterone, 10.4 ng/mL selenium, 25 μg/mL insulin, 0.8 μg/mL thyroxine, 0.6% glucose, and 6.6 mM glutamine, as reported previously [37,41,44]. AraC (5 µM; Sigma-Aldrich, San Luis, AZ, USA) was added to the cultures to inhibit proliferation and after 5–7 days in vitro (DIV), the cells were fixed for immunocytochemical detection of CNPase (1:200; Covance, Princeton, NJ, USA) and Olig-2 (1:200; Millipore, Burlington, NJ, USA). Finally, 10 random fields per coverslip were photographed under a Leica microscope using a20X objective and the proportion of CNPase^+^ cells was assessed relative to the respective controls (±s.e.m.).

### 2.9. Pharmacological Treatments

In order to study the mechanism underlying the activity of the FGF2/FGFR1/anosmin-1 system in specific aspects of cortical OPC biology (chemoattraction, motogenicity, proliferation, and differentiation), these processes were analyzed in the presence of the specific FGFR inhibitor SU5402 [21,29,34,43]. The SU5402 inhibitor was reconstituted in DMSO and administered at a final concentration of 10 µM, as used before [12,21,30,34,43,45,46,47]. A control of vehicle alone was used to account for any negative effects of DMSO on OPCs.

### 2.10. Differential FGFR Expression

FGFR1 expression in murine and human OPCs was assessed by dual immunofluorescence with the OPC marker NG2 (1:200; Millipore) and an antibody against FGFR1 (1:200; Santa Cruz Biotechnology, Dallas, TX, USA). In order to understand the response to FGF2 and anosmin-1 observed in OPCs from different ages, we studied the expression of different FGFRs: FGFR1, FGFR2, and FGFR3. Cells were plated in 96-well tissue culture plates (10,000 cells/well) and maintained at 37 °C in a 5% CO_2_ atmosphere and with 95% relative humidity in BS medium. After 1 DIV, the cells were fixed for 10 min at room temperature with 4% PFA. The OPCs were immunostained with antibodies against FGFR1 (1:200; Santa Cruz Biotechnology, Dallas, TX, USA), FGFR2 (1:50; Santa Cruz Biotechnology, Dallas, TX, USA), and FGFR3 (1:20; Santa Cruz Biotechnology, Dallas, TX, USA), and against tubulin (1:40,000; Sigma-Aldrich, San Luis, AZ, USA). Antibody binding was detected using 680 or 800 IRDye conjugated secondary antibodies and the plates were scanned using the Odyssey Infrared Imaging System (LICOR, Lincoln, NE, USA). Fluorescence intensity was measured according to the manufacturer’s instructions and the amount of FGFRs relative to the amount of tubulin was normalized to the FGFR expression in P0 samples. The values represent the mean (±s.e.m.).

### 2.11. Proliferation Assays

Purified OPCs were seeded on poly-l-lysine and laminin-coated coverslips (20,000 cells/coverslip), and FGF2 (20 ng/mL; R&D Systems, Minneapolis, MN, USA) or CT CHO or A1-expressing CHO cell extracts (0.1 mg/mL, see above) were added in BS medium. After 42 h in culture, a BrdU pulse (6 h) was administered and 24 h later, the cells were fixed for immunocytochemical detection of BrdU and Olig-2, as described previously [29]. Finally, 10 random fields per coverslip were photographed under a Leica microscope using a 20X objective and the data were expressed as the percentage of proliferating OPCs relative to the controls (±s.e.m.) considered as 100%.

### 2.12. 1H HR-MAS NMR Spectroscopy of OPCs

1H HR-MAS NMR measurements were performed on a Bruker spectrometer operating at 9.4 T (proton Larmor frequency of 400.14 MHz). The spectra were acquired at 4 °C to minimize the effect of temperature on cell stability during the acquisition time and to reduce the variations in some amino acids [48]. Sample spinning at the magic angle was applied at a speed of 2.4 kHz. The acquisition sequence and parameters were selected to assess the presence of metabolites of interest and their mobility, essentially using T2-filtered (CPMG) 1D experiments recorded with relaxation times of 2 and 60 ms. Low power presaturation during the interscan delay of 3 s was applied for water suppression. Typically, 256 scans were accumulated using a spectral width of 20 ppm, and the total acquisition time per spectrum was 13 min.

The spectra were processed using MestReNova version 8.1 software (Mestrelab Research, Santiago de Compostela, Spain) and all free induction decays were processed with exponential multiplication (0.5 Hz line-broadening) prior to Fourier transformation, followed by baseline correction. The chemical shifts were referenced as follows: a small amount (5 µL) of 10 mM DSS in D_2_O was added to one sample of intact cells and referenced to DSS = 0 ppm, after which all the spectra were processed identically and aligned (using the creatine methyl resonance at 3.026 ppm) with respect to the sample with added DSS. Of the various methods to estimate or measure the intensity of NMR signals [49], we compared peak intensity ratios using the methyl signal of creatine as an internal reference. Creatine is commonly used as an internal concentration reference for in vivo 1H NMR because its concentration correlates with the number of metabolically active cells and it can be used as a measure of viable cell number [50]. Cells were washed (three times) with a deuterated PB solution, centrifuged, and the cell pellet was frozen in liquid nitrogen and stored at −80 °C. Typically, approximately 60 μL of the cell pellet was placed into a 4 mm Ø zirconia rotor for each sample.

### 2.13. Imaging Analysis

Fluorescence digital images were obtained with a DFC480 FX digital camera (Leica, Wetzlar, Germany) coupled to a Leica DM5000 B microscope or using a SP5 resonant scanner (Leica Microsystems, Wetzlar, Germany). 

### 2.14. Statistical Analysis

For all experimental conditions, the results are represented as mean ± S.E.M. and were analyzed with ANOVA (for multicomparisons) and Students’s *t*-test (to compare just two groups) or the corresponding on rank tests in the case of non-parametric distributions, by using SigmaPlot 11 software (SPSS Science, Inc.) and critical values of: * *P* < 0.05, ** *P* < 0.01, and *** *P* < 0.001.

## 3. Results

### 3.1. Changes in the Intrinsic Properties of OPCs with Aging

**Figure 1:**
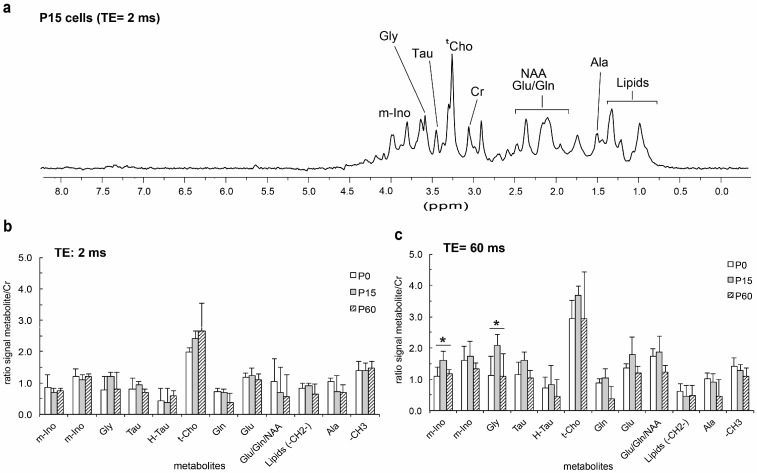
Representative 1H HR-MAS NMR spectra of intact Oligodendrocyte Precursor Cells (OPCs) with water suppression illustrating the chemical shift assignment of selected metabolites corresponding to a sample of OPCs (P15) and with a TE of 2 ms (**a**), and the intensities of main metabolites normalized with respect to creatine for TE of 2 ms (**b**) and 60 ms (**c**). An increase in the relative intensities of P15 metabolites with respect to those of P0 and P60 for the higher TE of 60 ms is observed, suggesting a decrease in cytoplasm viscosity. Error bars represent s.e.m. for n = 4. For all experimental groups the results were analyzed using One-Way ANOVA: * *P* < 0.05.

The intrinsic properties and metabolic pathways of murine OPCs isolated from the cerebral cortex were assessed at different ages (P0, P15, and P60). For the first time, we assessed these changes using proton high resolution-magic angle spinning nuclear magnetic resonance (1H HR MAS NMR) spectroscopy, which allows a large number of metabolites or small molecules in the cytoplasm to be systematically evaluated and their interaction with the local environment probed [48,49,50]. This approach may therefore provide valuable information on cellular physicochemical characteristics that may be correlated with cellular processes of interest. We obtained three spectra for each group of OPCs, with two different echo-times TE (“T2 filters”, TE: 2 and 60 ms), analyzing differences in signal intensities of peaks associated to the metabolites present in intact OPCs (see Table 2, Figure 1a). If we analyzed the proton spectra corresponding to P0, P15, and P60 for a short echo time (TE: 2 ms), we could see that there were not large differences between ages (Figure 1b). However, at TE of 60 ms, a relative increase in most peak intensities at P15 is observed (Figure 1c). At long echo times (TE: 60 ms), the intensity of the peaks associated with macromolecules or molecules with low mobility decreases (i.e., molecules bound to membranes or intracellular structures as lipids). It is remarkable that one of the most significant (*p* < 0.05) signal changes corresponds to myo-inositol, a metabolite known to be present in cell cytoplasm. Signal intensities depend on their T2 relaxation time, which is directly related to the molecular mobility in the cytoplasm. The higher metabolite mobility at P15 suggests a lower viscosity in the cytoplasm and a low interaction between metabolites and the cytoskeleton, which, as shown later, are associated with a decrease in migratory capability and a decrease in the proliferation rate. These results indicate that OPCs isolated at P15 display intrinsic differences to those isolated at P0 or P60.

**Figure 2:**
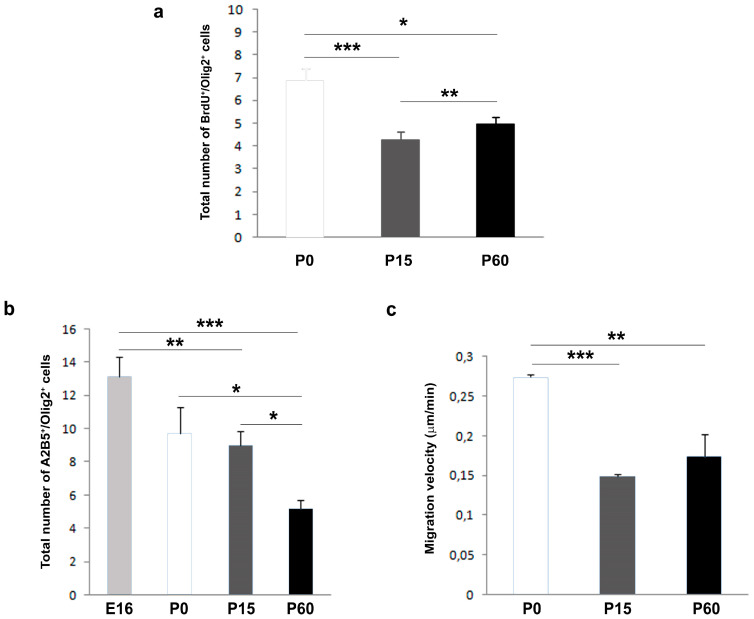
OPC proliferation and migration rates differ with age. (**a**) Proliferation rate of neonatal, postnatal and adult Oligodendrocyte Precursor Cells (OPCs). The number of proliferating OPCs also decreases with age. (**b**) Transmigrated cells of different ages after 20 h in control conditions: E16, P0, P15, and P60. The number of transmigrated cells decreases with age. (**c**) Histogram represents the migration speed at P0, P15, and P60. The migration speed is higher at P0, and it diminishes in OPCs from P15 and P60 mice. For all experimental groups, the results were analyzed using ANOVA and a Student´s *t*-test: * *P* < 0.05, ** *P* < 0.01, and *** *P* < 0.001.

Since the differences in the metabolic patterns may give rise to differences in other aspects of OPC behavior such as proliferation and migration, it was not surprising that the rate of proliferation was significantly higher at P0 and lowest at P15 (Figure 2a). Notably, OPCs isolated from adults (P60) proliferated at an intermediate rate (Figure 2a) and in general, there was a significant decrease in the migratory capacities of OPCs with age (Figure 2b). This was corroborated by video time-lapse analysis, where P15 and P60 OPCs migrated slower than perinatal OPCs (Figure 2c). Moreover, P60 OPCs stopped less often during their migration in control conditions (Table 3); P0 OPCs migrated further while P15 OPCs migrated over the shortest distance (P0, 332 μm; P15, 128 μm; P60, 210 μm). These data highlight the heterogeneity in OPC behavior (i.e., proliferation rate and migratory properties) under basal conditions, which could be related to differences in their metabolic pattern and variation in relaxation times of metabolites (i.e., cytoplasm viscosity). The highest levels in stops in control conditions were detected in P15 OPCs (Table 3), which may reflect the large-scale myelination that is ongoing at that time [51,52].

### 3.2. Migrating OPCs Respond Dynamically to FGF2 and Anosmin-1 in an Age-Dependent Manner

**Figure 3:**
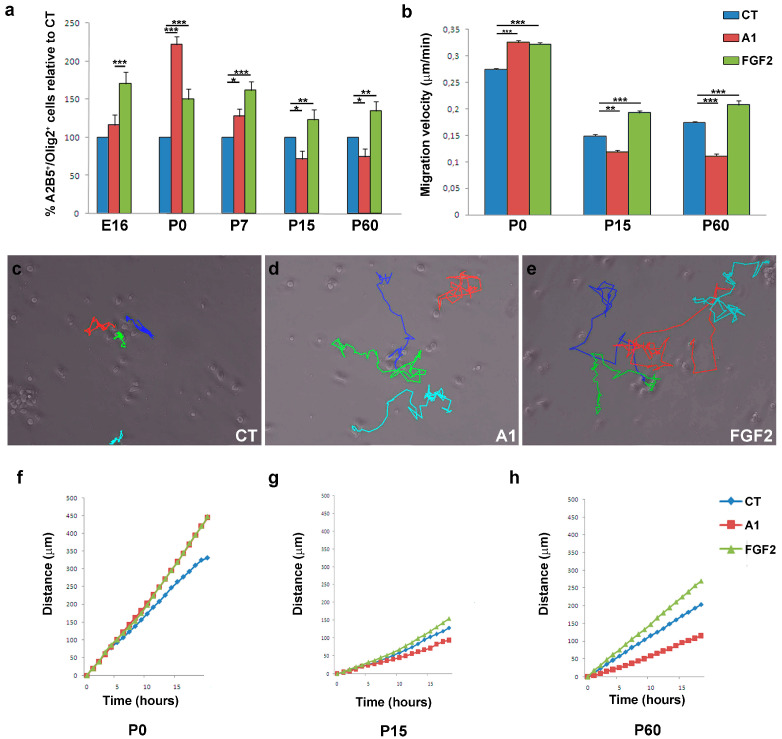
FGF2 and anosmin-1 control OPC migration. (**a**) Histograms showing the proportion of Oligodendrocyte Precursor Cells (OPCs) relative to the controls quantified in the presence or absence of FGF2 or anosmin-1 at different stages: E16, P0, P7, P15, and P60. ANOVA showed anosmin-1 had no effect on migration at E16 and P7 in contrast to other stages, while an effect of FGF2 was evident at all stages. (**b**) Histogram from the video time-lapse analysis representing the speed of migration at P0, P15, and P60 in the presence of either factor. (**c**–**e**) Images show the manual tracking at the end of the video time-lapse recording for P0 OPCs, in control conditions (**c**) and in the presence of anosmin-1 (**d**) or FGF-2 (**e**). (**f**–**h**) Analysis of the total distance migrated after 18 h in all conditions: P0 (**f**), P15 (**g**), and P60 (**h**). All experimental groups were analyzed with a Student´s *t*-test: * *P* < 0.05, ** *P* < 0.01, and *** *P* < 0.001.

FGF-2 and anosmin-1 exert distinct chemotropic effects on rat SVZ neuroblasts, and on rodent embryonic and postnatal OPCs, mainly acting via FGFR1 [12,21,29,34,43,45]. Hence, these molecules represent a good tool to systematically study the physiological heterogeneity in OPC populations, avoiding the variability in structure and origin by restricting our study to cerebral cortex OPCs. Exposure of OPCs isolated at different stages (E16, P0, P7, P15, and P60) to FGF2 homogeneously increased the number of transmigrating cells, confirming the previously described chemoattractive effect of this factor (and its potential motogenic effect as well: Figure 3a). The effect of FGF2 on migration was significantly stronger on OPCs isolated from E16 and early postnatal stages (P0 and P7) than on those from later stages, reflecting a gradual loss of sensitivity with age and CNS maturation. Interestingly, anosmin-1 induced different OPC responses depending on the age of the cells: neutral for E16 OPCs; significant chemoattraction of OPCs isolated at P0 and P7; and chemorepulsion in young adults (P15 and P60: Figure 3a). At all ages except P0, FGF2 triggered a stronger OPC response than anosmin-1 (Figure 3a).

To more clearly distinguish between the chemotropic and motogenic effects of FGF2 and anosmin-1 (direction or rate of migration), we followed OPCs from different aged mice by video time-lapse microscopy. FGF2 increased the motility of OPCs at all the stages studied and it increased the velocity of OPCs by around 20% relative to their counterparts in control conditions (Figure 3b). By contrast, anosmin-1 augmented the motility of P0 OPCs, yet it clearly reduced the speed of P15 and P60 OPCs (Figure 3b). Interestingly, P0 OPCs migrated further in the presence of either of the molecules tested than in control conditions, and they stopped less frequently (Table 3). On the other hand, P15 and P60 OPCs behaved similarly, migrating further and with fewer stops in the presence of FGF2, and anosmin-1 exerted exactly the opposite effects (Figure 3c–h; Table 3). Together, our results suggest that both FGF2 and anosmin-1 mainly influence the motility of OPCs (motogenic effects), which is consistent with previous data from our group [21,29].

### 3.3. Migration of Human OPCs in the Presence of FGF2 and Anosmin-1

**Figure 4:**
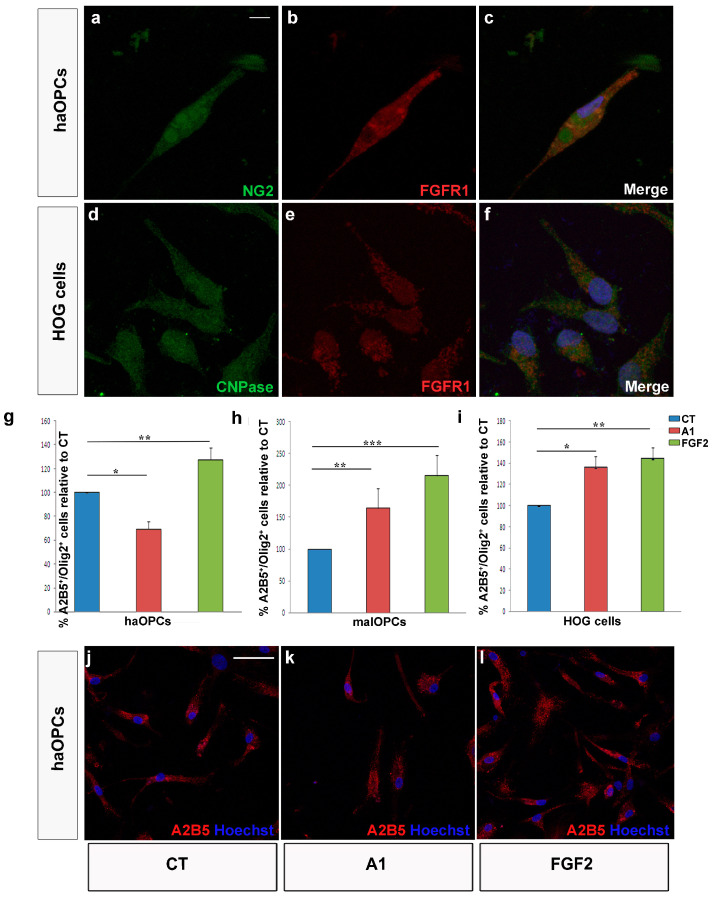
Effect of FGF2 and anosmin-1 on human Oligodendrocyte Precursor Cells (OPCs) migration. Adult human OPCs (haOPCs; (**a**–**c**) and Human OligodendroGlioma (HOG) cells (**d**–**f**) expressed FGFR1. In both types of tumor cell analyzed, malOPCs (**h**) and HOG cells (**i**), the response to FGF2 and anosmin-1 was the same. haOPCs responded to FGF2 like malOPCs, while the response to anosmn-1 is the opposite in these cell types (**g**). (**j**–**l**) Images of malOPCs in chemotaxis chambers stained with A2B5 (red). Scale bar represents 7.5 µm for a–f and 25 µm for j–l. The results obtained with Student´s *t*-test are represented as: * *P* < 0.05, ** *P* < 0.01, and *** *P* < 0.001.

**Figure 5:**
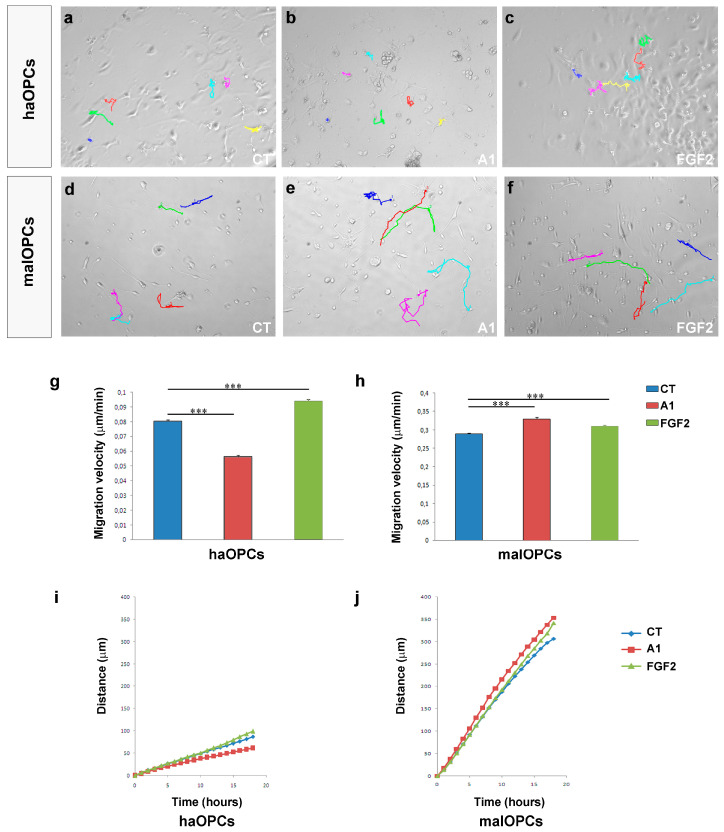
Video time-lapse imaging of human Oligodendrocyte Precursor Cells (OPCs) migration. (a–f) The manual tracking of haOPCs (**a**–**c**) and malOPCs (**d**–**f**). haOPCs migrate faster in the presence of FGF2 (**g**), while anosmin-1 (A1) has the opposite effect. malOPCs show the contrary pattern with a higher migration velocity for A1-treated cells and lower for FGF2-treated cells (**h**). Distance plots are represented in (**i**) and (**j**). Each colour in a-f represent the tracked path of one cell. The results of migration velocity with a Student´s *t*-test are represented as: *** *P* < 0.001.

As indicated above, molecules involved in the migration of OPCs during development are up-regulated in adults in conjunction with demyelination (including that occurring in MS lesions in humans), suggesting the need to test the effects of these molecules in adult OPCs. To complement this analysis, we sought to obtain information from human adult OPCs, evaluating the effects of both anosmin-1 and FGF2 on three kinds of human OPCs in chemotaxis chambers: an oligodendroglioma cell line (HOG cells), and on OPCs isolated from tumor tissue (malOPCs) and non-tumor tissue (haOPCs) obtained through neurosurgery (the exact origin of these samples is indicated in the Methods). We first confirmed that these three cell types expressed FGFR1, the receptor thought to mediate the effects of FGF2 and anosmin-1 on OPC migration (Figure 4a–f; [29,34,46,53,54]). It was not surprising to find that the malOPCs were more motile in control conditions than haOPCs (Table 3). However, exposure to the two factors tested also produced differences, whereby the number of transmigrated haOPCs significantly increased in the presence of FGF2 and was significantly lower in the presence of anosmin-1 (Figure 4g,j–l), a response similar to those of P60 mouse OPCs (Figure 3a). In addition, both proteins were motogenic and acted as chemoattractants for malOPCs and HOG cells (Figure 4h–l), as also reported for P0 murine OPCs (Figure 3a). It should be noted that the effect of anosmin-1 was stronger on malOPCs than on the immortalized cell line. Video time-lapse analysis of haOPCs revealed that anosmin-1 reduced their migration velocity while increasing the number of stops, the opposite effect to that observed with FGF2 for the same parameters (Figure 5a–j, Table 3), reflecting the contribution of pathological environment to the heterogeneity of OPC behavior. Together, these results from adult OPCs demonstrated that they are a heterogeneous population of cells, in which the physiological heterogeneity depends on the species and age. Indeed, this highlights the risk of directly extrapolating from drug testing on OPCs isolated from neonatal/early postnatal rodent brains to the human adult scenario.

### 3.4. The Biological Effects of FGF2 and Anosmin-1 on OPC Proliferation and Differentiation

**Figure 6:**
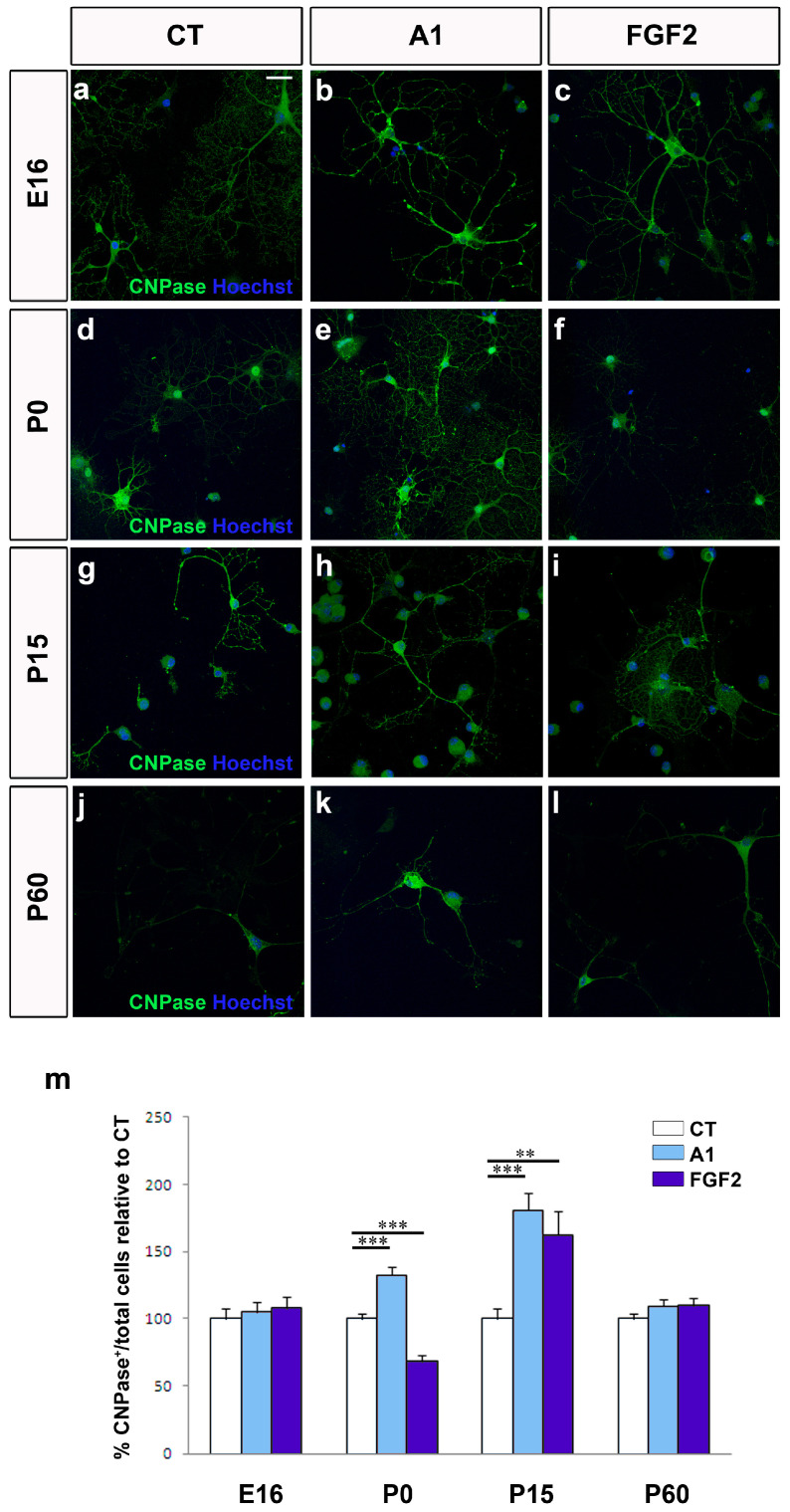
The effects of FGF2 and anosmin-1 on OPC differentiation. Images show mature oligodendrocytes from E16 (**a**–**c**), P0 (**d**–**f**), P15 (**g**–**i**) and P60 (**j**–**l**) mice labeled with CNPase (green) in control conditions and in the presence of FGF2 or anosmin-1. (**m**) Histogram represents the proportion of CNPase^+^ cells relative to the controls at all the stages analyzed. Scale bar represents 25 µm for a–l and the results were analyzed with a Student´s *t*-test: ** *P* < 0.01, and *** *P* < 0.001.

Rather than limiting our study to cell migration, we also studied how FGF2 and anosmin-1 affected OPC proliferation and differentiation. Both these factors had a constant effect on cell proliferation and while FGF2 significantly increased the number of newly generated OPCs at each developmental stage analyzed in young mature OPCs, anosmin-1 had no evident effect whatsoever on cell proliferation (Supplementary Figure S1a). Regarding OPC differentiation towards myelin-forming phenotypes, more heterogeneous results were obtained. In OPCs from embryonic (E16) and young adult (P60) stages, neither of these factors altered the rate of differentiation relative to the control conditions (Figure 6a–c,j–l,m). In contrast, FGF2 and anosmin-1 exerted opposite effects on P0 cells, while both these factors drove P15 OPCs towards a myelinating phenotype (Figure 6d–f,g–i,m).

### 3.5. The effects of FGF2 and Anosmin-1 on OPCs are FGFR-Dependent

**Figure 7:**
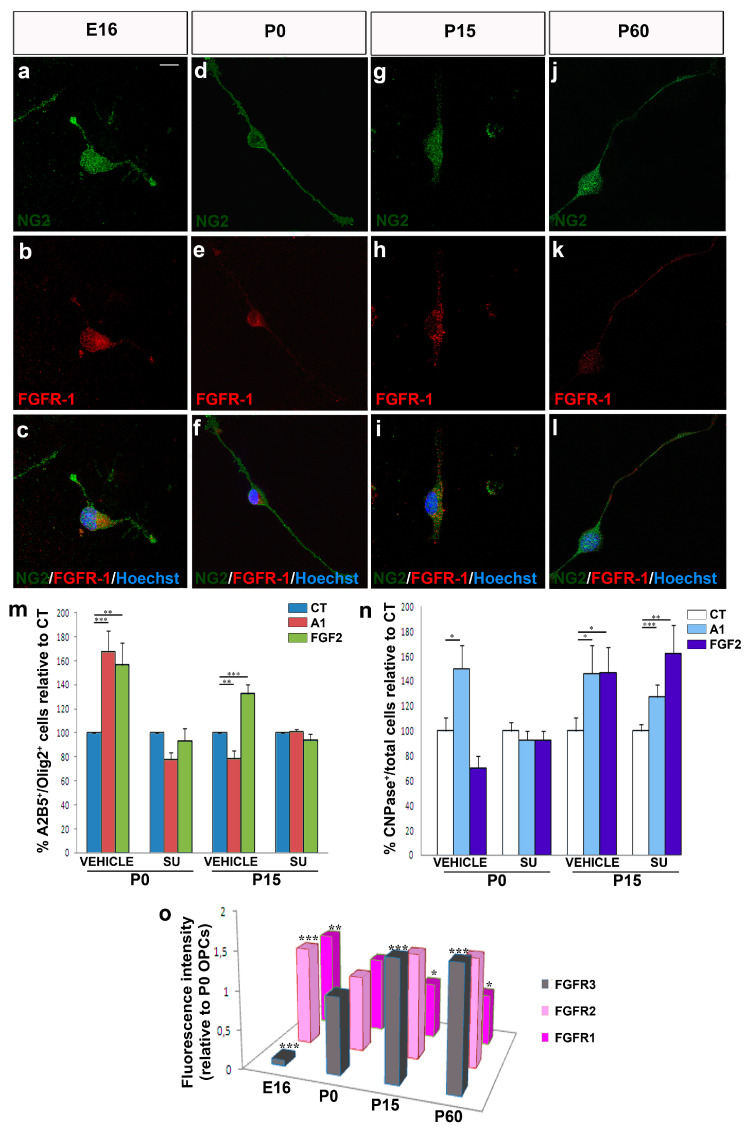
FGFR expression and its influence on OPCs. (**a**–**l**) FGFR1 is expressed by OPCs at all the stages analyzed: E16 (**a**–**c**), P0 (**d**–**f**), P15 (**g**–**i**), and P60 (**j**–**l**). (**m**) Histogram showing the results of chemotaxis at P0 and P15 in the presence of SU5402, which reverts the effects of anosmin-1 and FGF2. (**n**) CNPase^+^ cells at P0 and P15 in cultures treated with 10 µM of SU5402 or the vehicle alone (see Methods). No changes were observed in the presence of SU5402 for P0 but differences existed in P15. (**o**) FGFR1, R2, and R3 expression by OPCs isolated at E16, P0, P15, and P60. Scale bar represents 7.5 µm for a–c, g–i, and 10 µm for d–f, j–l. The results were analyzed with a Student´s *t*-test: * *P* < 0.05, ** *P* < 0.01, and *** *P* < 0.001.

FGF2 and its receptors have been implicated in numerous processes, including cell proliferation, migration, differentiation, and survival (for reviews see: [55,56,57]). Although we previously demonstrated that FGF2 and anosmin-1 exert their effects on OPC migration via FGFR1 [12,21,29,45,46], we analyzed the expression of the three FGFRs present in the oligodendroglial lineage [58,59] in our cultures of OPCs obtained from mice of different ages (E16, P0, P15, and P60). The FGFR1 was expressed by murine OPCs at every stage analyzed (Figure 7a–l). Thus, to confirm that FGFR1 influences OPC migration, we repeated the chemotaxis assays on P0 (both anosmin-1 and FGF2 acting as chemoattractants) and P15 OPCs (anosmin-1 no effect; FGF2 chemoattractant), in the presence or absence of an FGFR-signaling inhibitor (SU5402). This inhibitor abrogated both the chemoattraction and chemorepulsion provoked by FGF2 and/or anosmin-1, confirming FGFR1 as the FGF receptor involved in P0 and P15 OPC migration (Figure 7m).

In terms of OPC differentiation, SU5402 abolished the effects of both molecules at P0, reducing the percentage of CNPase^+^ cells to control levels, although it failed to achieve the same effect on P15 OPCs exposed to either FGF2 or anosmin-1 (Figure 7n). As FGF2 binds to every FGFR but anosmin-1 binds exclusively to FGFR1 [46,54], it suggests that other FGFRs expressed by OPCs at this stage are likely to be involved in their differentiation [58,59,60]. Finally, when we analyzed the effect of SU5402 on OPC proliferation, we noted that the increase in proliferation observed in the presence of FGF2 was abolished with the inhibitor (Supplementary Figure S1b).

Finally, we found a decrease in FGFR1 expression with age, which was most strongly expressed at the embryonic stage analyzed (Figure 7o). This gradual decline mirrors the decrease in migration speed and chemotaxis in the presence of FGF2 and anosmin-1 with age (Figure 1). In parallel, FGFR2 expression was weakest at P0, with no remarkable changes at the other three stages studied (Figure 7o). Finally, FGFR3 expression gradually increased with age, from very weak expression at E16 to an approximately 1.5-fold stronger expression at P15 and P60 than at P0 (Figure 7o), consistent with previous reports [59].

## 4. Discussion

Given their relative abundance in the adult CNS, their ability to move towards lesion sites, and their effectiveness in remyelination, there is ever-increasing interest in adult OPCs. However, this is a highly heterogeneous cell population and in practical terms, the capacity of these cells to remyelinate lesions is limited [13,21,23,61,62,63,64]. Different developmental signaling cues, including growth factors, are over-expressed locally in damaged areas, and they determine the survival, proliferation, migration, and correct differentiation of endogenous adult OPCs [20,21,25,26,65] (for a review see [66,67]). Here we systematically studied heterochronic OPCs isolated from the mouse cerebral cortex at E16, P0, P15, and P60, as well as OPCs isolated from samples of the adult human cerebral neocortex, avoiding the interference of the potential differences derived from the different developmental origins of the cells [2].

The heterogeneity in the responses of the OPCs also varied in function of the factor to which the cells were exposed. In response to FGF-2, OPCs responded in a more consistent manner when proliferation and migration were explored than when differentiation was studied. At each developmental stage studied, as well as in OPCs isolated from the cerebral cortex of adult mice and humans, FGF2 significantly enhanced cell proliferation (consistent with previous reports [56,68,69]) and certain migratory parameters, reflecting a motogenic and chemoattractive effect as reported previously [21,29,34,58,70]. It is interesting that the effect on human OPCs isolated from the safety-margins of tumors (malOPCs) was, in absolute numbers, stronger than that on human adult OPCs isolated from non-tumor samples (haOPCs) or even HOG cells (of human origin). It is also true that P15 OPCs had both a lower rate of proliferation and a poorer mobility than the OPCs isolated from P0 and P60 mice. Our data on OPC differentiation towards myelin-forming phenotypes substantially differed with age. As such, while FGF2 interfered with OPC differentiation of P0 OPCs (as indicated elsewhere [36,60]), it favored the differentiation of P15 OPCs and it had a neutral effect on OPCs isolated from the adult cerebral cortex, independent of the species (similar results were obtained with rat P0 OPCs—data not shown—and haOPCs). The results obtained by NMR spectroscopy on murine OPCs also identified different metabolic patterns for P15 compared to P0 and P60. This novel technique was previously used to analyze extracts of different cells types, including stem cells [71,72], although it was not applied to intact cells as occurred here. Our NMR results showed higher metabolite mobility for P15 cells, which suggests a lower viscosity in the cytoplasm and a lower interaction between metabolites and the cytoskeleton. The less viscous cytoplasm of P15 OPCs compared to P0 or P60 could explain the decrease in the migratory activity and proliferation OPCs at these stages [73,74,75].

In contrast to FGF2, anosmin-1 had no effect on OPC proliferation and it only potentiated OPC differentiation at early stages (P0, P15), having a neutral effect on differentiation at adult stages. However, the effects of anosmin-1 on OPC migration differed dramatically in function of both species and age, increasing the motility of P0 and tumor-related human OPCs (malOPCs and also the HOG cell line), while it slowed down the migration of P15 and P60/adult OPCs, and that of haOPCs. These latter experiments confirmed earlier data and to some extent they explain the factors that condition OPC responses to anosmin-1 [12,29,34].

With respect to the different effects observed with human OPCs and other tumor cells, anosmin-1 seems to promote/attract the migration of malOPCs and immortalized cell lines ([54,76,77,78,79]; data herein). This might contribute to cancer cell dispersion and metastasis, while anosmin-1 can contribute to the immobilization of cells in both physiological conditions (data herein) and in non-tumoral diseases like MS [21]. Nevertheless, it was suggested elsewhere that the *Anos1* gene (former *Kal-1* gene; [80]) would be a suppressor for other cancer types [78,81], which merits more systematic study. Likewise, the opposing or cooperative effects of FGF2 and anosmin-1 on tumor cells are still to be evaluated.

Undoubtedly, our study provides particularly interesting results in terms of the similarities and differences between OPCs isolated from the murine and human adult brain. The migratory properties of adult OPCs isolated from both species displayed parallel responses to FGF2 and anosmin-1, which is far from similar to the responses observed in early postnatal OPCs. Previous work showed that OPC heterogeneity depends on the anatomical region from which they are isolated and on the way they respond to injury [23,82,83]. Our data demonstrate that, in addition, it also depends on the species, the stage in the life cycle, and the functional system to which they pertain. This phenomenon should be taken into account in future studies that search for pharmacological agents to promote the motility of human OPCs isolated from different origins (the fetal or adult brain) in order to drive remyelination [67,84,85]. As such, and irrespective of their origin (murine or human), adult OPCs behave distinctly to OPCs isolated at earlier stages, at least in part because their metabolic profiles are very different. In addition, their response to different cues, such as FGF2 and anosmin-1, is modified with age. Although not as systematic as our current work, earlier data also suggest that the effects triggered by growth factors and motogenic cues (PDGF, FGF2) are more constant than those driven by chemotropic molecules and/or cytokines (semaphorins, netrin, anosmin-1, CCLs) [38,64,66,86]. In this regard, we have shown that remyelinating “small molecules” inhibiting phosphodiesterase-7 and GSK3 activities have very constant effects on OPCs in spite of the age or the species [41,87]. Hence, these data together probably reflect a hierarchy among the different pathways that influence the biology of OPCs during development and in different species. All these aspects of OPC biology should be taken into account in preclinical proofs-of-concept that would derive in successful clinical trials to obtain new remyelinating agents to treat MS [88].

In conclusion, the data presented here help understand the response of endogenous OPCs to lesions in the adult CNS. As such, therapeutic approaches for neurodegenerative diseases should focus on the potentiation of the endogenous OPCs present in the adult brain, enhancing their spontaneous remyelinating capacity. Accordingly, it should also be borne in mind that the modulation of FGF family members might also improve myelin repair in diseases like MS.

## Figures and Tables

**Table 1:** Summary of human samples used for in vitro experiments.

Patient	Age (Years)/Sex	Diagnostic	Lobe	Sample Weight (gr)
540	51/Female	Epilepsy	Temporal	4, 9
573	24/Male	Epilepsy	Temporal	2, 9
T-590	47/Female	Tumoral	Temporal	1, 9
554	50/Female	Epilepsy	Temporal	2, 6
574	45/Female	Epilepsy	Temporal	1, 4
559	45/Female	Epilepsy	Temporal	1, 1
579	50/Male	Epilepsy	Temporal	2, 6
603	53/Female	Epilepsy	Temporal	1, 1
549	32/Female	Epilepsy	Temporal	2
641	27/Female	Epilepsy	Temporal	1, 3
T-604	37/Female	Tumoral	Temporal	0, 7
363	51/Female	Epilepsy	Temporal	4
583	40/Male	Epilepsy	Temporal	0, 8
T-1-SC	62/Female	Tumoral	Temporal	0, 2
T-2-SC	68/Male	Tumoral	Temporal	0, 6
T-3-SC	61/Female	Tumoral	Temporal	1, 5
T-5-SC	51/Female	Tumoral	Temporal	3, 3

**Table 2:** Chemical shifts of main metabolites observed in Oligodendrocyte Precursor Cells (OPCs).

Metabolite	δ (ppm), Multiplicity, Number H
myo-inositol	3.93, m
myo-inositol	3.77, m
glycine	3.54, s 2H
taurine	3.42, t, 2H
hipotaurine	3.30, t, 2H
total-choline	3.23, s, 9H
glutamine	2.44, m, 2H
glutamate	2.34, m, 2H
glutamine/glutamate/N AA	2.12
acetate	1.91, s, 3H
lipids	1.70
alanine	1.47, d, 3H
lactate and lipids (methylene)	1.31
lipids and aminoacids (methyl)	0.94, t, 3H

**Table 3:** Analysis of OPC migration. The table shows the number of stops of migrating OPCs expressed as: number of stops/number of analyzed cells in control conditions and in the presence of FGF2 and anosmin-1.

	CONDITION Number of Stops/Number of Analyzed Cells		
Species and Age	CT	FGF2	Anosmin-1
Mouse P0	6.2	3.5	0.85
Mouse P15	11.4	1.52	20.7
Mouse P60	3.76	2.63	8.42
haOPCs, adult	3.61	8.8	17.6
malOPCs, adult	23.7	19.7	23.1

## Supplementary Materials

The following are available online at https://www.mdpi.com/2077-0383/9/6/1681/s1, Figure S1: Effects of FGF2 and anosmin-1 on OPC proliferation.

## Abbreviations

CNS	Central Nervous System
DMEM	Dulbecco’s Modified Eagle’s Medium
ECM	Extracellular Matrix
FBS	Fetal Bovine Serum
HRMAS	High-Resolution Magic Angle Spinning
MS	Multiple Sclerosis
OPC	Oligodendrocyte Precursor Cell
