# Supplementary material for: Functional Heterogeneity of Mouse and Human Brain OPCs: Relevance for Preclinical Studies in Multiple Sclerosis
Source: J Clin Med. 2020 Jun 2;9(6):1681. doi: 10.3390/jcm9061681 (PMC7355819; doi:10.3390/jcm9061681)

Supplementary Figure 1: Effects of Fibroblast Growth Gactor-2 (FGF-2) and anosmin-1 on Oligodendrocyte Precursor Cells (OPC) proliferation. (a) Quantification of the proliferating oligodendrocytes in the presence of FGF2 or anosmin-1 at the distinct ages studied. (**b**) Graph showing how SU5402 influences the effect of FGF2 on the proliferation of OPCs. The results were analyzed using a Student´s *t*-test: * *P* < 0.05, ** *P* < 0.01, and *** *P* < 0.001.

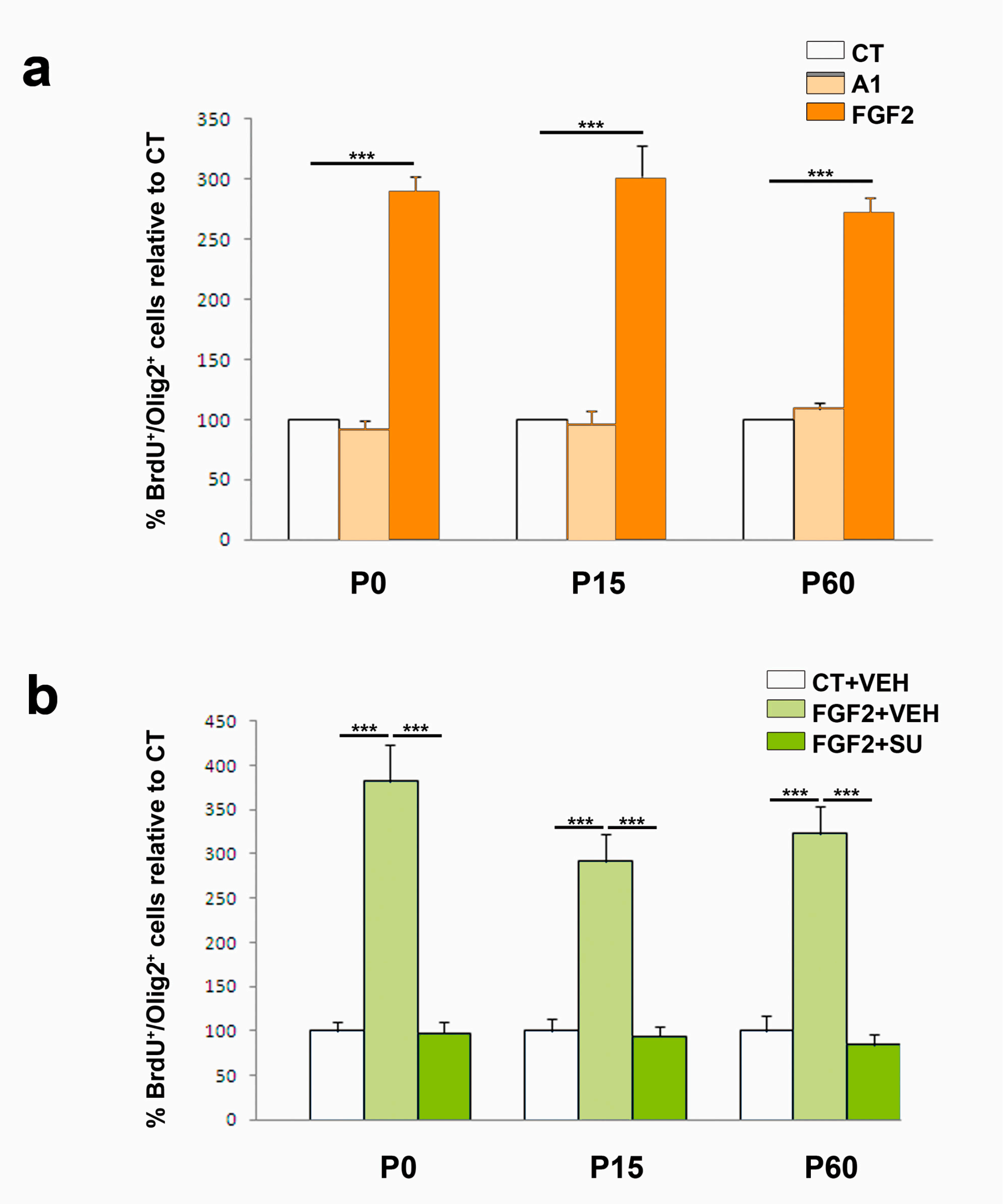

Supplement: Supplementary file 1 [file jcm-09-01681-s001.pdf]
